# A rare case of extramedullary plasmacytoma in the breast – an incidental finding in a 100-year-old following a fall

**DOI:** 10.1308/rcsann.2025.0068

**Published:** 2025-10-01

**Authors:** CC Gregory, RM Parks, N Srajagopal, E Gutteridge

**Affiliations:** Nottingham University Hospitals NHS Trust, UK

**Keywords:** Breast, Plasmacytoma, Multiple myeloma

## Abstract

Extramedullary plasmacytoma is a rare type of tumour consisting of monoclonal plasma cells found in soft tissues. Breast plasmacytomas account for less than 1% of all breast neoplasms. Breast plasmacytomas are challenging to identify on imaging due to nonspecific features. Their rarity as a cause of breast lump presents a management challenge to breast clinicians. Official guidance on management in the breast is lacking. We report a case of a 100-year-old woman who was diagnosed with multiple extramedullary plasmacytomas after an incidental finding of a right breast mass on a CT scan.

## Introduction

Extramedullary plasmacytoma is a rare type of tumour consisting of monoclonal plasma cells found in soft tissues, most commonly in the head and neck. Rarely they present in other sites, including the breast, testes, liver, spleen and pancreas.^1,2^

Extramedullary plasmacytoma can remain symptomatically silent for years; however, there is a risk of progression to multiple myeloma (MM) if left untreated.

We report a case of a 100-year-old woman who was diagnosed with multiple extramedullary plasmacytomas after an incidental finding of a right breast mass on a computed tomography (CT) scan.

## Case history

A 100-year-old woman presented with a right breast lump, which had been detected incidentally on a trauma CT scan during presentation to hospital with a fall. The patient underwent CT thorax, abdomen and pelvis as part of her initial investigations, which demonstrated multiple soft tissue densities in addition to the breast mass, including an omental density and bilateral adrenal masses, measuring 48mm on the right and 19mm on the left. The densities were thought to be neurofibromatosis, or an unusual presentation of metastatic breast cancer.

Following recovery from the fall, the patient attended the breast unit. On examination, there was a palpable 30×27mm malignant-feeling mass in the right central breast at the 3 o’clock position. There were no overlying skin changes, nipple discharge or other abnormalities associated with the breast. The right axilla, left breast and axilla were clinically normal.

The patient denied noticing any breast lump but reported some weight loss over the last year and reduced appetite. She had no previous breast or relevant family history. Past medical history included hypertension, chronic kidney disease (CKD) stage 4, type two diabetes mellitus (diet controlled), diabetic retinopathy, cataract surgery, osteoarthritis and oesophagitis. She was a nonsmoker and did not take any regular medications. She presented in a wheelchair, was independent with activities of daily living, and her Abbreviated Mental Test score was 10/10 and World Health Organisation performance status was 3.

Ultrasound scan confirmed a lobulated 33.7mm solid mass suspicious for malignancy and was subjected to core biopsy. Due to frailty she was not a candidate for mammogram. Recent blood tests were within normal limits, with an estimated glomerular filtration rate of 21 in keeping with CKD and did not show evidence of hypercalcaemia.

**Figure 1:**
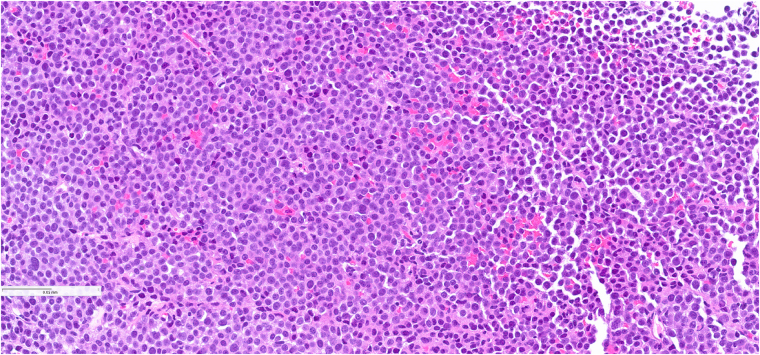
Hematoxylin and eosin stain shows the neoplastic plasma cells arranged in diffuse sheets, with condensed chromatin and abundant cytoplasm.

Histology revealed features consistent with a plasmacytoma. Breast tissue was replaced by diffuse sheets of cells with moderately pleomorphic nuclei, variably condensed chromatin and small nucleoli with plentiful cytoplasm. Some cells contained Dutcher bodies and many cells were noted to have Golgi zones (Figure 1). There was a moderately high proliferative fraction (approximately 50% Ki-67 positive).

**Figure 2:**
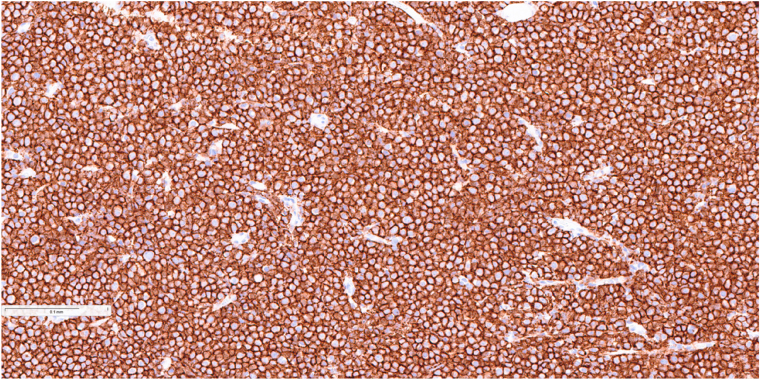
Immunohistochemical studies show the neoplastic plasma cells are expressing CD138 diffusely and strongly.

The mass was positive for tumour markers CD79a, CD138, MUM1, Bcl-2 and kappa light chain (Figure 2). It was negative for CD3, CD20, ER, PR, chromogranin, synaptophysin, CK5/6, SMA, p63, HER2, AE1/AE3, GATA3, E-cadherin, S100, HMB45, Melan-A, CD10, p53, BCL6, cyclin D1, CD56, c-Myc and PAX5.

The patient was referred to the myeloma multidisciplinary team (MDT) and a diagnosis of multiple extramedullary plasmacytomas was made. The patient had elevated serum free Kappa LC (55.47mg/l) while serum free Lambda LC was within normal limits (18.85mg/l). Her Kappa:Lamba ratio was also raised (2.94).

The patient declined any further invasive investigation or active treatment, including surgical excision and chemotherapy, and instead was offered standalone steroid therapy for appetite.

The patient passed away four months after her initial presentation at clinic from pneumonia and gastroenteritis.

## Discussion

Plasmacytoma is a rare type of tumour, originating from abnormal proliferation of a single plasma cell. It is associated most commonly with MM yet can occur in the absence of the disease or as a precursor. It can arise from within the bone as an osseous plasmacytoma, accounting for the majority of cases (70%), or in the soft tissues as an extramedullary plasmacytoma (30% of cases).^3^ The mechanism by which plasmacytoma appears in soft tissue includes haematogenous spread from bone in advanced MM, or can originate de novo at the site driven by local factors. It can occur as a solitary primary lesion, or uncommonly as multiple extramedullary plasmacytomas as with our patient.^4^

The most common sites of extramedullary plasmacytoma include the upper aerodigestive tract: oropharynx, tonsils, lung, mediastinum, liver, pancreas, stomach, mesentery, kidneys, and the small and large bowel.^5^ Haloui *et al* also reported extramedullary plasmacytoma in the ovaries.^6^

Reports of extramedullary plasmacytoma in the breast are incredibly rare. Breast plasmacytomas represent just 1.5% of all diagnosed plasmacytomas, and equally account for less than 1% of all breast neoplasms.^1^ Although most commonly unilateral, bilateral breast plasmacytomas have been observed in patients with extensive MM.^7,8^ They can occur at any age – the youngest reported case in the medical literature is 37 years old and the oldest is 79 years.^1,9^ To the best of the authors’ knowledge, this is the first case of breast plasmacytoma in a patient more than 80 years of age and the only case in a centenarian.

Breast plasmacytomas are challenging to identify on imaging due to their nonspecific features. They can present sonographically as heterogeneous, both hyperechoic and hypoechoic, hypervascular, and ill-defined lesions.^5^ When presenting in the breast, triple assessment with biopsy and histopathology is therefore essential.

Although extramedullary plasmacytoma can remain undetected for years, if left untreated it can progress into MM.^1^ This risk is much lower in patients with extramedullary plasmacytoma, however, (10–15%) compared with those with osseous plasmacytoma (50–60%). The five-year survival rate is relatively broad, at between 40% and 80%.^10^

Its rarity as a cause of breast lump presents a management challenge to breast clinicians. An MDT-based approach comprising radiologists, surgeons and haematologists is necessary for optimal patient care. Recommended treatment for solitary plasmacytoma is radiotherapy and surgical excision.^11^ However, official guidance on management of breast plasmacytoma is lacking. Consideration of the size and location of the lesion will guide surgical excision and radiotherapy. More extensive disease may require systemic treatments including chemotherapy. The age and comorbidity status of the patient will guide the overall management strategy.

## Conclusions

Breast plasmacytomas are challenging to diagnose and manage. They can present incidentally, as our case shows, and remain undetected due to their asymptomatic presentation, yet if left untreated may progress into MM. They are not easily identified with imaging, therefore histopathology is fundamental. A paucity of guidance on management of extramedullary plasmacytoma in the breast means an MDT approach is essential.

Patients who present with a breast lump and have a background of MM should be considered for extramedullary plasmacytoma as a rare differential.
